# Analytical solution of *l-i SEIR* model–Comparison of *l-i SEIR* model with conventional *SEIR* model in simulation of epidemic curves

**DOI:** 10.1371/journal.pone.0287196

**Published:** 2023-06-14

**Authors:** Xiaoping Liu

**Affiliations:** Department of Medicine, Department of Neuroscience, Rockefeller Neuroscience Institute, West Virginia University Health Science Center, Morgantown, West Virginia, United States of America; Villanova University, UNITED STATES

## Abstract

The Susceptible-Exposed-Infectious-Recovered (*SEIR*) epidemic model has been commonly used to analyze the spread of infectious diseases. This 4-compartment (*S*, *E*, *I* and *R*) model uses an approximation of temporal homogeneity of individuals in these compartments to calculate the transfer rates of the individuals from compartment *E* to *I* to *R*. Although this *SEIR* model has been generally adopted, the calculation errors caused by temporal homogeneity approximation have not been quantitatively examined. In this study, a 4-compartment *l-i SEIR* model considering temporal heterogeneity was developed from a previous epidemic model (Liu X., Results Phys. 2021; 20:103712), and a closed-form solution of the *l-i SEIR* model was derived. Here, *l* represents the latent period and *i* represents the infectious period. Comparing *l-i SEIR* model with the conventional *SEIR* model, we are able to examine how individuals move through each corresponding compartment in the two *SEIR* models to find what information may be missed by the conventional *SEIR* model and what calculation errors may be introduced by using the temporal homogeneity approximation. Simulations showed that *l-i SEIR* model could generate propagated curves of infectious cases under the condition of *l*>*i*. Similar propagated epidemic curves were reported in literature, but the conventional *SEIR* model could not generate propagated curves under the same conditions. The theoretical analysis showed that the conventional *SEIR* model overestimates or underestimates the rate at which individuals move from compartment *E* to *I* to *R* in the rising or falling phase of the number of infectious individuals, respectively. Increasing the rate of change in the number of infectious individuals leads to larger calculation errors in the conventional *SEIR* model. Simulations from the two *SEIR* models with assumed parameters or with reported daily COVID-19 cases in the United States and in New York further confirmed the conclusions of the theoretical analysis.

## Introduction

*SIR* (Susceptible-Infectious-Recovered) and *SIR*-derived epidemic mathematical models (such as *SEIR* model; Susceptible-Exposed-Infectious-Recovered) are commonly used in the analysis of transmission of infectious diseases [1–6]. These models have been playing an important role in formulating the proper social interventions to slow down the spread of COVID-19 [7–15]. The *SEIR* model is a 4-compartment model. In the conventional *SEIR* model, it assumes that a susceptible individual in the compartment *S* may enter compartment *E* after the individual is exposed to an infectious source; an exposed individual in compartment *E* may enter compartment *I* at a certain rate and become an infectious individual; and an infectious individual in compartment *I* may enter compartment *R* at a certain rate and become a recovered individual. Using the four variables *S*(*t*), *E*(*t*), *I*(*t*) and *R*(*t*) to represent the numbers of individuals in the four compartments at time *t* respectively, the relationship between the four variables is defined by the following differential equations [2]:

$$\frac{dS(t)}{dt}=-\frac{\beta S(t)I(t)}{N},$$ (1A)

$$\frac{dE(t)}{dt}=\frac{\beta S(t)I(t)}{N}-\sigma E(t)$$ (1B)

$$\frac{dI(t)}{dt}=\sigma E(t)-\gamma I(t),$$ (1C)

$$\frac{dR(t)}{dt}=\gamma I(t)$$ (1D)

In Eq (1), *N* is the total population; and the coefficients *β*, *σ* and *γ* are constants. One of the underlying assumptions for the above equations is: the change in *E*(*t*), *I*(*t*) or *R*(*t*) at time *t* is determined by a fraction of *E*(*t*) and *I*(*t*) (*σE*(*t*) and *γI*(*t*)) at time *t*. This means that after a susceptible individual in compartment *S* becomes an exposed individual in compartment *E* at time *t*, this newly exposed individual is able to enter compartment *I* at the same rate *σE*(*t*) as all other pre-existing individuals in compartment *E*, without chronological order. However, the reality is different: after a susceptible individual is exposed to the infectious source and becomes an exposed individual in compartment *E*, the newly exposed individual cannot become an infectious individual and enter compartment *I* until the exposed individual passes a latent period (*l* days on average). Likewise, an infectious individual in compartment *I* can only recover after an infectious period (*i* days on average). Thus, a *l-i* chronological order is an inherent relationship that exists among the 4 variables, whereas the conventional *SEIR* model uses an approximation of temporal homogeneity to calculate the transfer rate of infected individuals moving from compartment *E* to *I* and from *I* to *R*. Some people may ask, what calculation errors will the conventional *SEIR* model make when simulating the epidemic data? In this study, we developed a 4-compartment *l-i SEIR* model from the 3-compartment *l-i AIR* model published previously [16, 17]. The *l-i SEIR* model, like the *l-i AIR* model, has considered temporal heterogeneity of individuals in the model. The analytic solution of the *l-i SEIR* model in closed-form was derived. The 4 compartments in the *l-i SEIR* model have the same meaning as the 4 compartments in the conventional *SEIR* model. Both the *l-i SEIR* model and the conventional *SEIR* model have the same series of 4 compartments from the compartment *S* to *E* to *I* to *R*. Thus, we are able to examine how individuals move through each corresponding compartment in the two *SEIR* models to find what information the conventional *SEIR* model may miss and what calculation errors in the conventional *SEIR* model may result from using the temporal homogeneity approximation.

### Theory and methods

#### *l-i SEIR* model

The *l-i SEIR* model is developed from a recently published *l-i AIR* model based on the following three assumptions:

1. During the spread of an infectious disease, the decreasing rate of the number of susceptible individuals on day *n*, (*S*_*n*-1_—*S*_*n*_), equals to the number of infectious individuals (*I*_*n*_) times the ratio of *S* to *N* (*S*_n-1_*/N*) and multiplies a rate coefficient *β*_*n*_ ($\beta_{n}I_{n}\frac{S_{n-1}}{N}$). This assumption is the same as the one described in the classic *SIR* model. Here, *N* is the number of susceptible people right before the infectious disease spread out. If all people in the population are susceptible to the infectious agents before the infectious disease spreads out, *N* equals to the total population *P*. However, if a portion of people has immunity to the infectious disease before the infectious disease spreads out, *N* is smaller than *P*. It should be emphasized that the impact of other interventions including social distancing, face mask requirements and quarantine measures on the transmission rate of infectious diseases is factored into the coefficient *β*_*n*_. Because the policies and requirements of these interventions may change during the transmission process of an infectious disease, *β*_*n*_ is a time-dependent coefficient in the *l-i SEIR* model.
2. When an individual in compartment *S* enters compartment *E* as a newly exposed individual, this individual will stay in compartment *E* for a latent period (*l* days on average) before entering compartment *I* as a newly infectious person. The newly infectious person will stay in compartment *I* for an infectious period (*i* days on average) before entering compartment *R*. The total time length of the latent period and the infectious period is *c* (*c* = *l*+*i*).
3. Only a fraction *α* of individuals (*I*_*n*_) in compartment *I* are confirmed and reported as daily new cases on day n (*y*_*n*_). In our calculations in this paper, we will use the 7-day average of *y*_*n*_, which is written as ${\bar{y}}_{n}$.

We can derive the following recursive Eq (2A) from Assumption (1), write the following recursive Eqs (2B)–(2D) based on Assumption (1) and (2), and give the following Eq (2E) based on Assumption (3).


$$S_{n}-S_{n-1}=-\beta_{n}I_{n}\frac{S_{n-1}}{N}$$ (2A)

$$E_{n}-E_{n-1}=\beta_{n}I_{n}\frac{S_{n-1}}{N}-(S_{n-l-1}-S_{n-l})=(S_{n-1}-S_{n})-(S_{n-l-1}-S_{n-l})$$ (2B)

$$I_{n}-I_{n-1}=(S_{n-l-1}-S_{n-l})-(S_{n-c-1}-S_{n-c})$$ (2C)

$$R_{n}-R_{n-1}={(S}_{n-c-1}-S_{n-c})$$ (2D)

$$y_{n}=\alpha I_{n}\approx {\bar{y}}_{n}$$ (2E)

Regarding the initial conditions of the above equations, we assume:

- For all *n*<0 before the infectious disease starts to spread, *E*_*n*_ *= I*_*n*_ *= R*_*n*_ *=* 0 and *S*_*n*_ = *N*.
- At *n* = 0, the first person in compartment *S* is exposed to the infectious source and enters compartment *E*. Thus, we have *S*_0_ = *N*-1, *E*_0_ = 1, and *I*_0_ = *R*_0_ = 0.

Furthermore, it should be noted that the impact of different interventions on the transmission rate of infectious diseases is achieved by changing the coefficient (*β*_*n*_ in Eq (2A)) of the transmission rate of infectious diseases. When we use *l-i SEIR* model to analyze COVID-19 cases in the US, *β*_*n*_ can be considered a composite coefficient contributed from all states in the US. Thus, each state, when an intervention measure is performed, can make a certain change to the value of *β*_*n*_ in the US more or less depending on population of the state, type of the intervention measures, and other related factors. The time-dependent value of *β*_*n*_ in the US was determined by fitting *αI*_*n*_ (or *y*_*n*_) in Eq (2E) to the reported daily new cases (${\bar{y}}_{n}$) in the US. Similarly, the time-dependent value of *β*_*n*_ in the state of New York (NY) was also determined by fitting *y*_*n*_ in Eq (2E) to the reported daily new cases (${\bar{y}}_{n}$) in NY. To compare the *l-i SEIR* model with the conventional *SEIR* model in the simulations of daily COVID-19 cases, we assume that the rate coefficient *β* in Eqs (1A) and (1B) is a time dependent coefficient that can be written as *β*(*t*). Furthermore, the following equation, which is similar to Eq (2E) in the *l-i SEIR* model, should be added into Eq (1) for determining *β*(*t*) from the reported daily COVID-19 cases by fitting *αI*(*t*) to ${\bar{y}}_{n}$.


$$y(t_{n})=\alpha I(t_{n})\approx {\bar{y}}_{n}$$ (1E)

In Eq (1E), *t*_*n*_ represents the time on the nth day.

#### Analytical solution of Eqs (2A)–(2D)

From Eqs (2A) and (2B) we can derive:

$$\begin{array}{l} E_{n}=E_{n-1}+{(S}_{n-1}-S_{n})-(S_{n-l-1}-S_{n-l})=E_{n-1}+{(S}_{n-1}-S_{n-l-1})-(S_{n}-S_{n-l})\\ =[E_{n-2}+{(S}_{n-2}-S_{n-l-2})-(S_{n-1}-S_{n-l-1})]+{(S}_{n-1}-S_{n-l-1})-(S_{n}-S_{n-l})\\ =E_{n-2}+{(S}_{n-2}-S_{n-l-2})-(S_{n}-S_{n-l})\\ =E_{0}+{(S}_{0}-S_{-l})-(S_{n}-S_{n-l})=1+(-1)-(S_{n}-S_{n-l})=S_{n-l}-S_{n} \end{array}$$ (3A)

By observing how the red subscript “*n*-1” changes to “*n*-2” in the related terms during the above derivation, we can easily deduce the final terms in Eq (3A) when the subscript is reduced to 0. From the initial conditions, we know that *E*_0_ = 1 and *S*_0_ –*S*_-*l*_ = (*N*-1)-*N* = –1 in Eq (3A). Thus, we can simplify the final expression for *E*_n_.

Similar to the derivation of expression for *E*_n_, we have:

$$\begin{array}{l} I_{n}=I_{n-1}+(S_{n-l-1}-S_{n-l})-(S_{n-c-1}-S_{n-c})=I_{n-1}+(S_{n-l-1}-S_{n-c-1})-(S_{n-l}-S_{n-c})\\ \;=[I_{n-2}+(S_{n-l-2}-S_{n-c-2})-(S_{n-l-1}-S_{n-c-1})]+(S_{n-l-1}-S_{n-c-1})-(S_{n-l}-S_{n-c})\\ \;=I_{n-2}+(S_{n-l-2}-S_{n-c-2})-(S_{n-l}-S_{n-c})=I_{0}+(S_{-l}-S_{-c})-(S_{n-l}-S_{n-c})\\ \;=0+0-(S_{n-l}-S_{n-c})=S_{n-c}-S_{n-l} \end{array}$$ (3B)

From Eq (2D) we have:

$$\begin{array}{l} R_{n}=R_{n-1}+(S_{n-c-1}-S_{n-c})=[R_{n-2}+(S_{n-c-2}-S_{n-c-1})]+(S_{n-c-1}-S_{n-c})\\ \;=R_{n-2}+(S_{n-c-2}-S_{n-c})=R_{0}+(S_{-c}-S_{n-c})=0+(N-S_{n-c})=N-S_{n-c} \end{array}$$ (3C)

Substitution of Eq (3B) into Eq (2A) gives:

$$S_{n}=S_{n-1}-\beta_{n}I_{n}\frac{S_{n-1}}{N}=S_{n-1}(1-\beta_{n}\frac{I_{n}}{N})=S_{n-1}(1-\beta_{n}\frac{S_{n-c}-S_{n-l}}{N})$$

Thus, the final solution of *S*_*n*_ is the following formula:

$$\begin{array}{l} S_{n}=S_{n-1}(1-\frac{\beta_{n}(S_{n-c}-S_{n-l})}{N})=S_{n-2}(1-\frac{\beta_{n-1}(S_{n-c-1}-S_{n-l-1})}{N})(1-\frac{\beta_{n}(S_{n-c}-S_{n-l})}{N})\\ \;=S_{0}\prod_{m=1}^{n}\left(1-\frac{\beta_{m}(S_{m-c}-S_{m-l})}{N}\right) \end{array}$$ (3D)

Finally, we obtain the following analytical solutions of the variables *S*_*n*_, *E*_*n*_, *I*_*n*_ and *R*_*n*_ from Eqs (3A)–(3D):

$$S_{n}=S_{0}\prod_{m=1}^{n}\left(1-\frac{\beta_{m}(S_{m-c}-S_{m-l})}{N}\right)$$ (4A)

$$E_{n}=S_{n-l}-S_{n}$$ (4B)

$$I_{n}=S_{n-c}-S_{n-l}$$ (4C)

$$R_{n}=N-S_{n-c}$$ (4D)

The above closed-form solution, Eq (4), can be easily validated by comparing the calculated *R*_*n*_ from the two *l-i* models when they have the same set of parameters *l*, *i*, *N*, and *β*_*n*_. From Eq (4A), we can calculate the value of *S*_*n*_ on any day *n* if *l* and *c* are known and *β*_*n*_ is a constant. Then, values of *E*_*n*_, *I*_*n*_ and *R*_*n*_ can be calculated from Eqs (4B)–(4D) after *S*_*n*_ is determined. If *β*_*n*_ varies with time, *β*_*n*_ on any day *n* can be determined by fitting *αI*_*n*_ to ${\bar{y}}_{n}$ based on Eq (2E) assuming that *α* has been determined as described previously [18]. Before starting the fitting process, we need to plot the calculated *y*_*n*_, *y*(*t*_*n*_) and the reported ${\bar{y}}_{n}$ into one graph in the Excel program, and thus, we can visualize how *y*_*n*_ or *y*(*t*_*n*_) changes with respect to ${\bar{y}}_{n}$ while we regulate the rate coefficient *β*_*n*_ or *β*(t), respectively, during the fitting process. In this case, we need Eqs (4A), (4C) and (2E) to determine *S*_*n*_, *I*_*n*_ and *β*_*n*_, and then find *E*_*n*_ and *R*_*n*_ from Eqs (4B) and (4D).

### Differences in the definition of rate change of variables *E*, *I* and *R* between *l-i SEIR* model and conventional *SEIR* model

Both the conventional *SEIR* model and the *l-i SEIR* model use the average latent period (*l*) and the average infectious period (*i*) to determine the rate of change in model variables *E*, *I* and *R*. The main difference between the two *SEIR* models is about how to use *l* (or *σ =* 1*/l*) and *i* (or *γ* = 1/*i*) to calculate the rate of change in *E*, *I* and *R*. This difference can be clearly seen by comparing Eq (1) with Eq (2). For example, both Eq (1B) and Eq (2B) are used to determine the rate of change of *E*(*t*) or *E*_*n*_ with respect to time *t* or day *n*, respectively. Here, *E*(*t*) or *E*_*n*_ is the number of people in the latent period. Both Eq (1B) and Eq (2B) have the following form:

$$v_{E}=v_{E\_in}-v_{E\_out}$$ (5)

**Fig 1:**
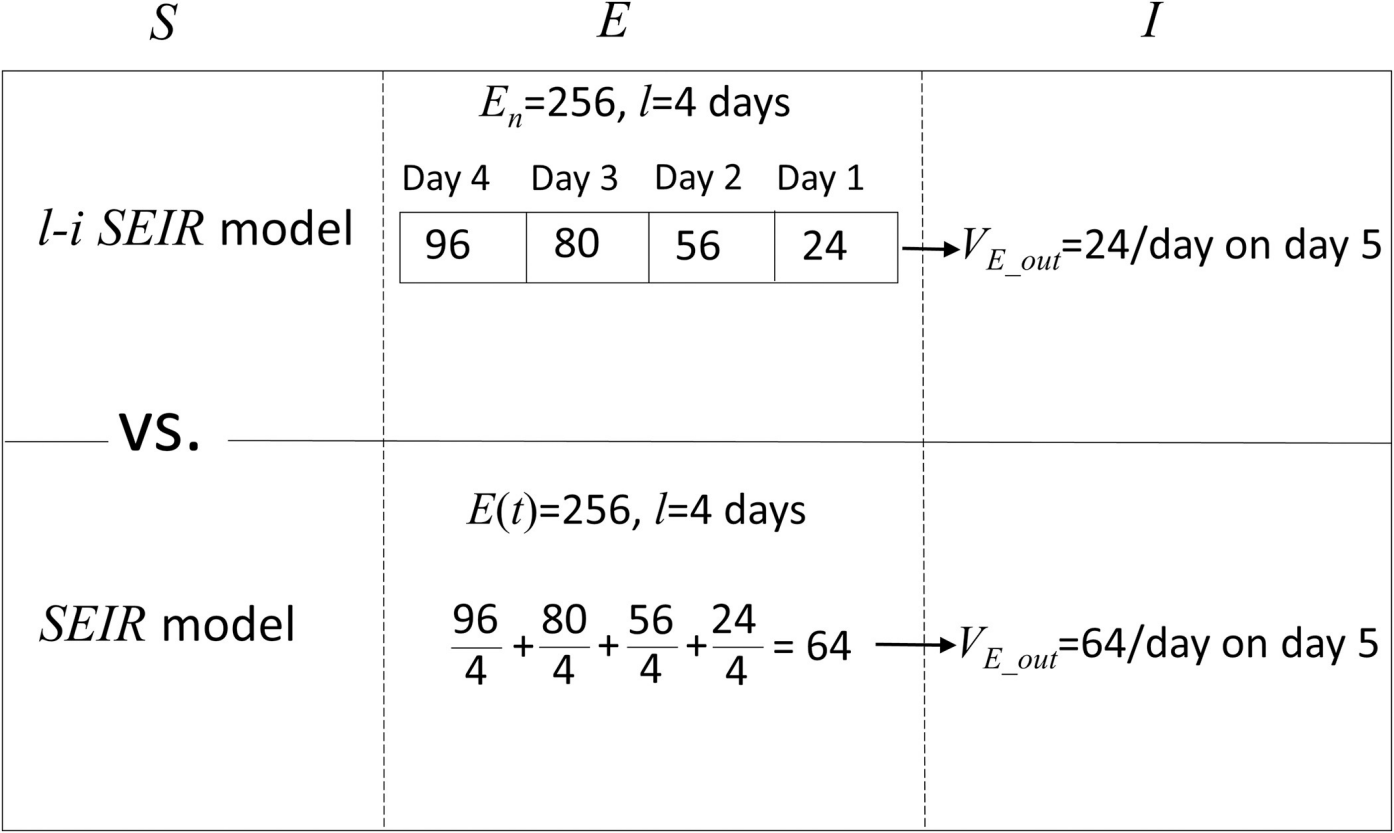
Comparison of difference between the l-i SEIR model and the conventional SEIR model in finding *v*_*E*_*out*_, the number of people who leave compartment E and enter compartment I per unit time.

Here, *v*_*E*_ is the rate of change in *E*(*t*) or *E*_*n*_, which is written as *dE*(*t*)/*dt* in Eq (1B) or (*E*_*n*_−*E*_*n*−1_) in Eq (2B). The term *v*_*E*_*in*_ is the number of people who enter compartment *E* per unit time, which is written as $\frac{\beta S(t)I(t)}{N}$ in Eq (1B) and ($\frac{\beta_{n}S_{n-1}I_{n}}{N}$) in Eq (2B). So far, there is no difference between Eq (1B) and Eq (2B) except that the former uses the differential form while the latter uses the difference form. The main difference between the two equations appears in the term *v*_*E*_*out*_. Based on the conventional *SEIR* model, if the number of people in compartment *E* is *E*(*t*) and the average latent period for each person is *l* days, then the number of people who leave compartment *E* per day can be defined as *E*(*t*)/*l* or $v_{E\_out}=\frac{E(t)}{l}=\sigma E(t)$. The method using this definition to find *v*_*E_out*_ sounds reasonable and is easy to perform in the calculation program. However, this definition ignores temporal heterogeneity from the initial stage of infection to the recovery stage of the infectious disease among the individuals (or samples) in compartment *E* and *I*. Is there any other reasonable and easy-to-implement definition, which has taken the temporal heterogeneity of samples into account, for calculating *v*_*E_out*_? To answer this question, let us make some assumptions (Fig 1). (1) There are currently 256 people in compartment *E* at the beginning of the morning of day 5 since the infectious disease started and spread. (2) The average latent period for each person is 4 days. (3) Among the 256 people in compartment *E*, 24 were infected (entered compartment *E*) on day 1, 56 on day 2, 80 on day 3 and 96 on day 4. Because we have known that the latent period *l* is 4 days and 24 people were infected on day 1, all these 24 people will transfer from compartment *E* (latent period) to compartment *I* (infectious period) on day 5 after they passed their 4 days of latent period. Thus, based on Eq (2B) of the *l-i SEIR* model, *v*_*E_out*_ on day 5 is 24/day. It is easy to understand that *v*_*E_out*_ on day 6 is 56/day because 56 infected people entered compartment *E* on day 2 (or 4 days ago). Simply speaking, the *l-i SEIR* model considers this temporal heterogeneity of samples and applies the principle of “first-in, first-out” to find *v*_*E_out*_. In comparison, the conventional *SEIR* model uses approximation of temporal homogeneity to find the rate of changes in *E*(*t*), *I*(*t*) and *R*(*t*) in (Eqs (1B)–(1D)) without considering the chronological order of samples in each compartment. The calculated *v*_*E_out*_ from Eq (1B) of the conventional *SEIR* model is 64/day (256/4) on day 5. Among the 64 people, 24 (96/4), 20 (80/4), 16 (56/4) and 6 (24/4) people come from those who entered compartment *E* on day 4, 3, 2 and 1, respectively. It can be seen that *v*_*E_out*_ calculated from Eq (1B) of the conventional *SEIR* model includes infected people in compartment *E* for all time frames from day 1 to day 4 because any person in compartment *E* has the same probability of moving from compartment *E* to compartment *I*, regardless of the chronological order of the person entering compartment *E*. In the above example, the number of daily new infections in compartment *E* increases with time from day 1 to day 4. In this rising phase of infection cases, the *v*_*E_out*_ on day 5 calculated from the conventional *SEIR* model Eq (1B) is 64/day. However, only 24 people in compartment *E* have passed their latent period on day 5, so the *v*_*E_out*_ determined from the *l-i SEIR* model Eq (2B) is 24/day. Thus, the conventional *SEIR* model overestimates *v*_*E_out*_ in the rising phase of infection cases. Clearly, if the rising rate of infection cases is greater, then the calculation error of *v*_*E_out*_ from the conventional *SEIR* model will be greater. Vice versa, it is not difficult to understand that if the number of daily new infections in compartment *E* decreases with time, the *v*_*E_out*_ calculated from the conventional *SEIR* model Eq (1B) will be smaller than the *v*_*E_out*_ determined from the *l-i SEIR* model Eq (2B). Therefore, the conventional *SEIR* model underestimates *v*_*E_out*_ in the falling phase of infection cases. Also, if the falling rate of infection cases is greater, then the calculation error of *v*_*E_out*_ from the conventional *SEIR* model will be greater. In the same way, we can discuss the differences in the definition of the rate change of variables *I* and *R* between *l-i SEIR* model and the conventional *SEIR* model.

### Calculation methods and programs

Simulations and calculations of model variables *S*, *E*, *I*, *R* and *y* were based on the analytical solution of the *l-i SEIR* model Eq (4) with Eq (2E) (or Eq (2)) and the conventional *SEIR* model Eq (1). The parameters *l*, *i* and coefficient *α* in *l-i SEIR* model equations for the simulations of the COVID-19 transmission in the United States and NY were determined with the methods described in previous studies [17, 18]. All calculation programs were written in Excel [19, 20]. COVID-19 data in the US and NY obtained from Worldometer website (https://www.worldometers.info/coronavirus/country/us/) and Wikipedia website (COVID-19 pandemic in New York (state)—Wikipedia), respectively.

## Results and discussion

### The conventional *SEIR* model can’t generate propagated epidemic curves when *l>i* like the *l-i SEIR* model

**Fig 2:**
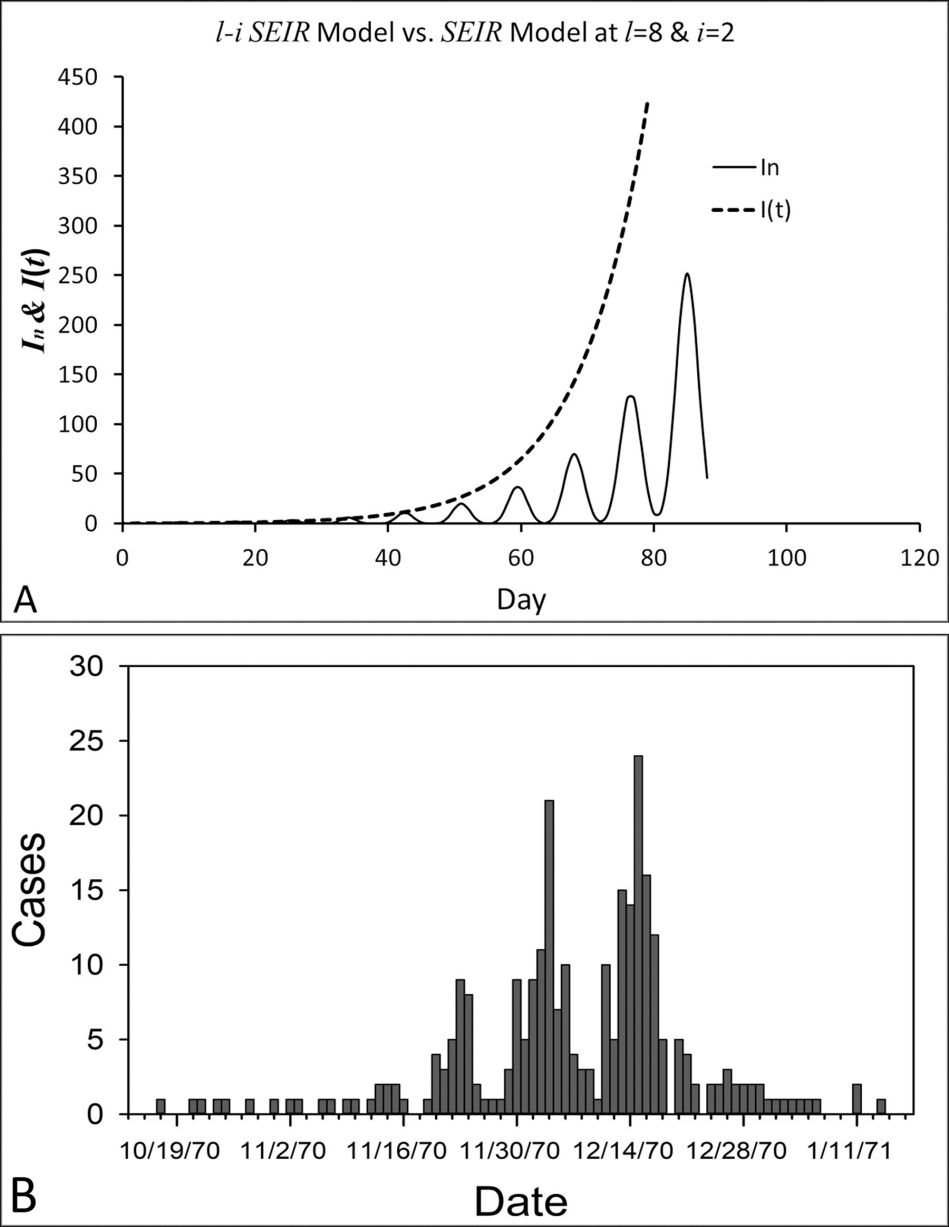
Comparison of the number of infectious individuals (*I*_*n*_) calculated from the *l-i SEIR* model with the number of infectious individuals (*I*(*t*)) calculated from the conventional *SEIR* model when *l>i*. Assuming that the latent period *l* = 1/*σ* = 8 days, the infectious period *i* = 1/*γ* = 2 days, *β*_*n*_ = *β*(*t*) = 1 and *N* = 3.3x10^8^, the *l-i SEIR* model generated a propagated epidemic curve for *I*_*n*_ (solid line in A), but the conventional *SEIR* model generated an epidemic curve for *I*(*t*), which increased in a near-exponential form (dashed line in A). Daily measles cases (an example of propagated epidemic curves) were reported in Aberdeen, South Dakota, USA from October 15, 1970 to January 16, 1971 (B).

In Fig 2, we compared the number of infectious individuals *I*_*n*_ calculated from the *l-i SEIR* model with the one calculated from the conventional *SEIR* model assuming *l*>*i* (Fig 2). It can be seen that the *l-i SEIR* model generated a propagated epidemic curve for the number of infectious individuals *I*_*n*_ as *l*>*i* by assuming *l* = 1/*σ* = 8, *i* = 1/*γ* = 2, *β*_*n*_ = *β*(*t*) = 1 and *N* = 3.3x10^8^ (the solid line in Fig 2A). However, under the same conditions (*l* = 1/*σ* = 8, *i* = 1/*γ* = 2 and *N* = 3.3x10^8^), the conventional *SEIR* model generated an epidemic curve, which increased in a near-exponential form, for the number of infectious individuals *I*(*t*) (the dashed line in Fig 2A). The one in Fig 2B shows the daily measles cases (an example of propagated epidemic curves) reported in Aberdeen, South Dakota, USA from October 15, 1970 to January 16, 1971 [21].

From Fig 2A, we can see that if *l*>*i*, then the *I*_*n*_ and *I*(*t*) curves, representing the number of infectious individuals, calculated from the two models, are largely different. The *l-i SEIR* model can generate a propagated epidemic curve (solid line in Fig 2A) that is similar to the reported propagated epidemic curve in Fig 2B. In contrast, the conventional *SEIR* model (dashed line in Fig 2A) does not generate a propagated epidemic curve even if the average latent period (1/*σ* = 8) is greater than the average infectious period (1/*γ* = 2). Why the *l-i SEIR* model can but the conventional *SEIR* model cannot simulate the propagated curve when latent period is longer than the infectious period? If we assume that the latent period *l* = 10 days, the infectious period *i* = 2 days, and that 10 infected people are all currently in the infectious period (compartment *I*), and they lead to 20 new people being infected during the 2 days of infectious period, then we will only see a peak with 10 cases of infectious people in the current infectious period. The reason is that the newly infected 20 people are still in the latent period, and we can only see the next peak with the 20 new cases after the 10 days of latent period. Keeping transmission in this way, we will observe a propagated epidemic curve. This result indicates that the chronological order of latent period-infectious period should be applied to epidemic models for assuring the chronological relationship among the model variables.

### Calculation errors generated from the conventional *SEIR* model increase with the rate of change in the number of infection cases

**Fig 3:**
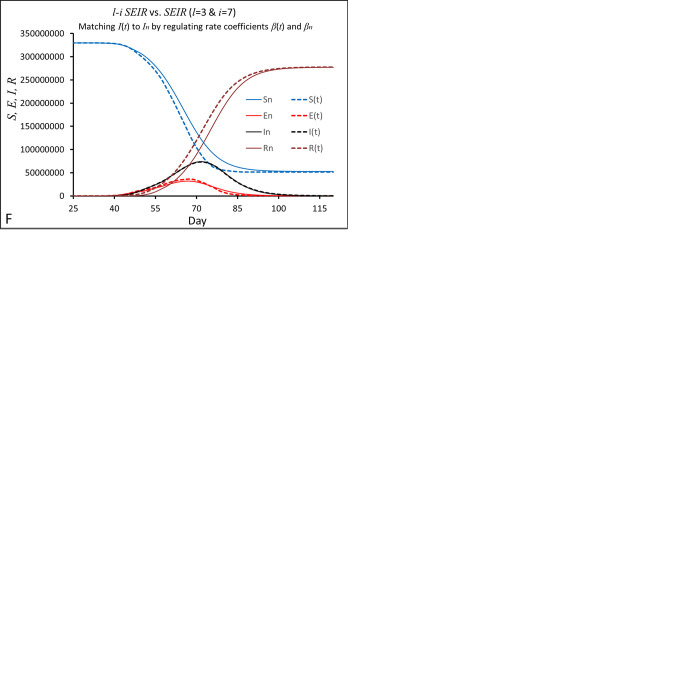
Examination of differences between curves *S*(*t*), *E*(*t*), *I*(*t*) and *R*(*t*) of the conventional *SEIR* model and their corresponding curves *S*_*n*_, *E*_*n*_, *I*_*n*_ and *R*_*n*_ of the *l-i SEIR* model at different rates of change in the number of infection cases. Simulations in (A)-(D) were performed by assuming parameters *β*_*n*_ = *β*(*t*) = 1 and *N* = 3.3x10^8^, and (A) *l* = 1/*σ* = 2, *i* = 1/*γ* = 8, (B) *l* = 1/*σ* = 3, *i* = 1/*γ* = 7, (C) parameters same as those in (B), but the initial date (the day on which the first person was infected) in the conventional *SEIR* model was postponed by 3 days, and (D) *l* = 1/σ = 4, *i* = 1/*γ* = 5, the initial date in the conventional *SEIR* model was postponed by 6 days. (E) Simulations were performed by assuming parameters *N* = 3.3x10^8^, *l* = 1/*σ* = 3, *i* = 1/*γ* = 7, and downregulating both *β*_*n*_ and *β*(*t*) to slow down *I*(*t*) and matching *I*(*t*) and *I*_*n*_ to each other. (F) Parameters are the same as those used in (*E*), but *I*(*t*) and *I*_*n*_ were further slowed down by reducing *β*_*n*_ and *β*(*t*).

The above theoretical analysis on the conventional *SEIR* model shows that if the rising rate of infection cases is greater, the calculation error of *v*_*E_out*_ from the conventional *SEIR* model will be greater. To test this outcome of theoretical analysis, we examined how the differences in the model variables *S*, *E*, *I* and *R* between the two *SEIR* models varied with the rate of change in the number of infection cases by regulating parameters and transmission coefficients in the two *SEIR* models. To change the rate of infection cases in the simulations, we set *β*_*n*_ = *β*(*t*) = 1, *N* = 3.3x10^8^ in Fig 3A–3D, but set *l* = 1/*σ* = 2 & *i* = 1/*γ* = 8 in Fig 3A; *l* = 1/*σ* = 3 & *i* = 1/*γ* = 7 in Fig 3B, 3C; and *l* = 1/*σ* = 4 & *i* = 1/*γ* = 5 in Fig 3D. In Fig 3E & 3F, we set *l* = 1/*σ* = 3, *i* = 1/*γ* = 7 with varied *β*_*n*_ and *β*(*t*). Under these conditions, both *SEIR* models generated sigmoidal curves for model variables *S* and *R*, and bell-shaped curves for model variables *E* and *I*. The curve *S*(*t*) (blue dashed line) is very close to the curve *S*_*n*_ (blue solid line) as *l* = 1/*σ* = 2, *i* = 1/*γ* = 8 (Fig 3A), and the peak positions of *E*(*t*) and *I*(*t*) are very close to the peak positions of *E*_*n*_ and *I*_*n*_ respectively. However, it should be pointed out that the time course of change in *R*(*t*) (purple dashed line) is significantly different from the time course of change in *R*_*n*_ (purple solid line): *R*(*t*) increases earlier and faster than *R*_*n*_ in the rising phase of *I*(*t*) approximately before the *I*(*t*) peak and increases slower than *R*_*n*_ in the falling phase of *I*(*t*) approximately after the *I*(*t*) peak. This result indicates that due to ignoring chronological order, the conventional *SEIR* model does overestimate (or underestimate) the transfer rate from compartment *E* to *I* and from *I* to *R* in the rising (or falling) phase of *I*(*t*).

In comparison to those parameters used for simulating the curves in Fig 3A, if we change parameters *l* (= 1/*σ*) to 3 and *i* (= 1/*γ*) to 7, we can see larger differences between the model variables (*S*_*n*_, *E*_*n*_, *In*, *Rn*) in *l-i SEIR* model and their corresponding variables (*S*(*t*), *E*(*t*), *I*(*t*), *R*(*t*)) in *SEIR* model (Fig 3B). However, these apparently large differences can be partially reduced by simply moving the initial date (the day on which the first person is infected) in the conventional *SEIR* model forward or backward by a few days. As shown in Fig 3C, the differences in model variables (*S*, *E*, *I* and *R*) between the two *SEIR* models become similar to those shown in Fig 3A after postponing the initial date of the conventional *SEIR* model by 3 days. Carefully comparing Fig 3C with 3A, we can know that the peak height, rising rate before the peak, and falling rate after the peak of *I*(*t*) and *I*_*n*_ in Fig 3C are smaller than those of *I*(*t*) and *I*_*n*_ in Fig 3A. Correspondingly, *R*(*t*) is closer to *R*_*n*_ in Fig 3C than in Fig 3A. If assuming *l* = 1/*σ* = 4, *i* = 1/*γ* = 5 and postponing the initial date of the conventional *SEIR* model by 6 days, the shapes of the simulated curves *S*, *E*, *I* and *R* in Fig 3D are similar to what we have seen in Fig 3A and 3C, but the peak height, rising rate before the peak, and falling rate after the peak of *I*(*t*) and *I*_*n*_ in Fig 3D further decrease compared to those in Fig 3A and 3C. Correspondingly, *R*(*t*) is closer to *R*_*n*_ in Fig 3D than in both Fig 3A and 3C. When using the same parameters as those used in Fig 3D, but decreasing the transmission rate *β*, we can further reduce *I*(*t*) and *I*_*n*_ and their rising rates as shown in Fig 3E & 3F. Correspondingly, *R*(*t*) is further close to *R*_*n*_ in Fig 3E & 3F comparing to that in Fig 3D.

To quantify these results from Fig 3A–3F, we made the following two definitions:

1. The normalized maximal calculation error in *R*(*t*), which is defined as (*R*(*t*)-*R*_*n*_)_max_/(*R*_*n*_)_max_. Here, (*R*(*t*)-*R*_*n*_)_max_ is the maximal difference between *R*(*t*) and *R*_*n*_, and (*R*_*n*_)_max_ is the maximal *R*_*n*_ within the time range considered. The normalized maximal calculation error will be used to check the closeness between *R*(*t*) and *R*_*n*_ calculated from the two *SEIR* models.
2. The relative rising rate, which is roughly inversely proportional to the time required to go from the bottom of *I*(*t*) (at ~5% of *I*(*t*)) to the top of *I*(*t*). For example, the bottom to top time of *I*(*t*) in Fig 3A is 10 days, so the relative rising rate of *I*(*t*) is 1/10 or 0.1 per day.

The factor (*R*(*t*)-*R*_*n*_) in the formula given in the above definition (1) is used to obtain the difference between *R*(*t*) of the conventional *SEIR* model ignoring chronological order of infected individuals in the model and *R*_*n*_ of the *l-i SEIR* model taking into account of the chronological order of individuals in the model. Thus, this formula is appropriate to examine the calculation errors caused by ignoring the chronological order of individuals in the conventional *SEIR* model. Because the chronological order of infected individuals also exists in the transmission process of infectious diseases in the real world, the calculation errors of the conventional *SEIR* model resulted from ignoring the chronological order of infected individuals also exist in the analysis of epidemic data in the real world.

The relative rising rates of *I*(*t*) and the normalized maximal calculation errors of *R*(*t*) obtained from Fig 3 are listed in Table 1.

**Table 1:** The relative rising rate of *I*(*t*) and the normalized maximal calculation errors of *R*(*t*).

Bottom to top time of *I*(*t*) (day)	Relative rising rate of *I*(*t*) (1/day)	Normalized Maximal calculation error of *R*(*t*)	Data Source and parameters (*l*, *i*, *β*) used in simulations
10	0.1000	0.2887	Fig 3A (2, 8, 1)
11	0.0909	0.2096	Fig 3G (2, 6, 1), (Fig not shown)
13	0.0769	0.1978	Fig 3C (3, 7, 1)
15	0.0667	0.1311	Fig 3D (4, 5, 1)
22	0.0455	0.1223	Fig 4C & 4D (4, 10, varied β)
85	0.0118	0.0586	Fig 4A & 4B (4, 10, varied β)
18	0.0556	0.1474	Fig 3E (3, 7, varied β)
24	0.0417	0.1140	Fig 3F (3, 7, varied β)

### Comparison of *l-i SEIR* model with the conventional *SEIR* model in simulations of COVID-19 transmission in the Unites States and in NY

**Fig 4:**
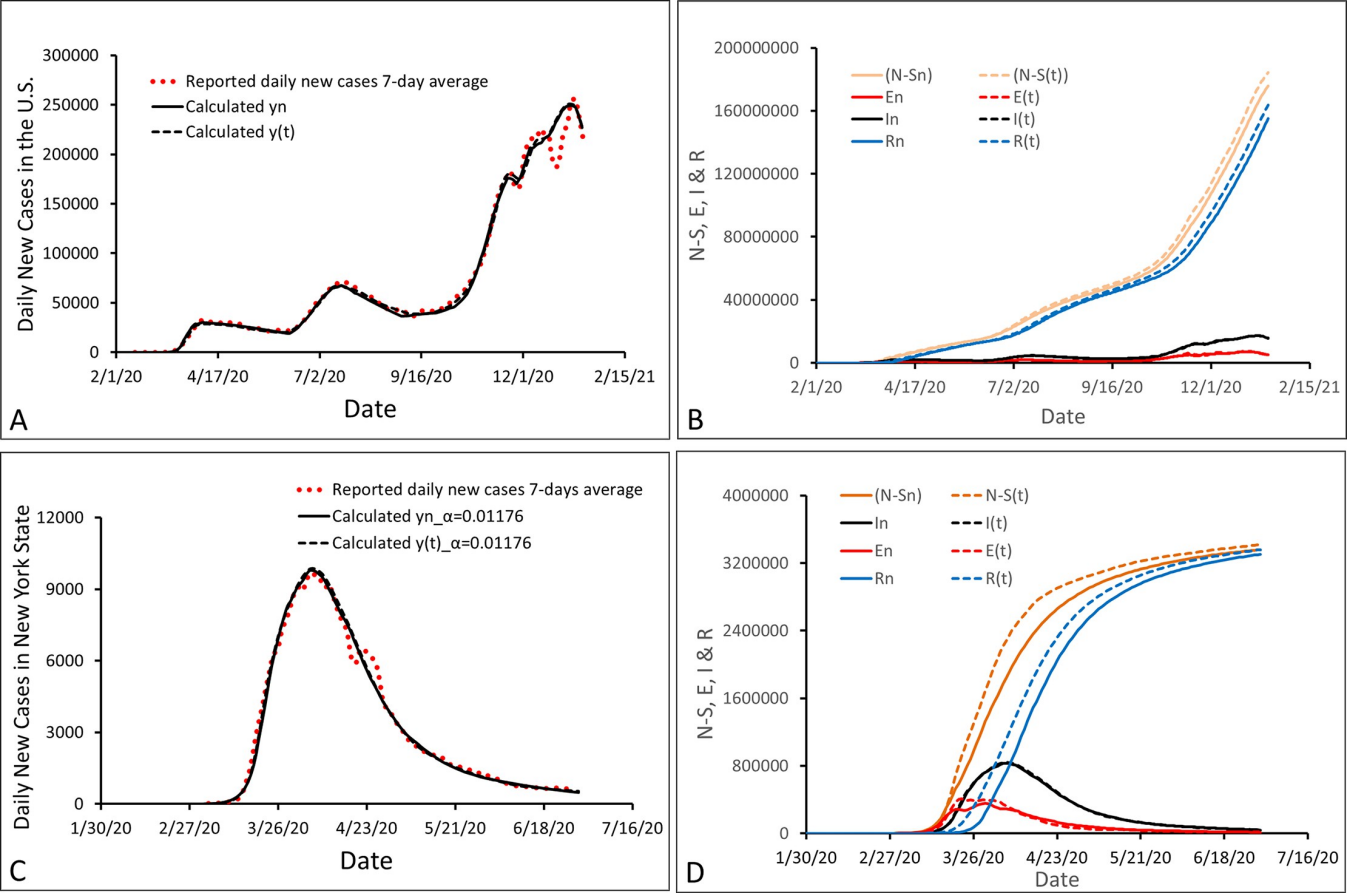
Comparisons of *l-i SEIR* model with conventional *SEIR* model in simulations of COVID-19 epidemic data in the US and in NY. (A) Fitting the numbers of daily new COVID-19 cases (*y*_*n*_ and *y*(*t*)) calculated from the two *SEIR* models to the numbers (red dots) of daily new COVID-19 cases ${\bar{y}}_{n}$ reported in the United States. (B) The calculated *S*, *E*, *I* and *R* curves from the two *SEIR* models after the fitting process in (A) was completed. (C) and (D) are the same as (A) and (B) except that the numbers of daily new COVID-19 cases ${\bar{y}}_{n}$ were reported in NY.

To compare the two *SEIR* models in the simulations of epidemic data in the real world, we separately applied the differential equations of the conventional *SEIR* model (Eq (1)) and the analytical solution (Eq (4) with Eq (2E)) or the recursive equation (Eq (2)) of *l-i SEIR* model to simulate the number of daily COVID-19 cases in the United States (reported before mid-January 2021) and in NY in 2020 (reported before the end of June, 2020) (Fig 4A–4D). Because many social intervention measures were performed for reducing the rate of COVID-19 transmission, the actual rate coefficient (*β*(*t*) and *β*_*n*_) of COVID-19 transmission in the United States within certain time frames may be much smaller than 1. As a result, the rate of COVID-19 transmission (Eqs (1A) and (2A)) and the number of infectious cases (*I*(*t*) and *I*_*n*_) were greatly reduced. Thus, it is interesting to examine whether we would see a significant reduction in calculation errors of the conventional *SEIR* model compared to the *l-i SEIR* model. We previously determined the parameters (*l*, *i* and α) in *l-i AIR* model for the simulations of daily COVID-19 cases in the United States [18] and NY [17]. Because *l-i SEIR* model was developed from *l-i AIR* model, the parameters *l*, *i* and α in *l-i SEIR* model are the same as those in *l-i AIR* model when the two *l-i* models are used in simulating the same set of epidemic data in the real world. In all of the simulations below, we let *l* = 4, *i* = 10 [17, 18]; and let *α* = 0.01453 [18] for the simulation of COVID-19 cases in the US and *α* = 0.01176 [20] for the simulations of COVID-19 cases in NY. The time-dependent *β*_*n*_ in *l-i SEIR* model was determined by regulating the value of *β*_*n*_ day after day for fitting the calculated *y*_*n*_ or *αI*_*n*_ (black solid line) to the reported ${\bar{y}}_{n}$ (red dots) based on Eq (2E) (Fig 4A and 4C). To find suitable sets of parameters for the conventional *SEIR* model in the simulations of daily COVID-19 cases in the US and NY, we first tried to use the same sets of parameters as those used in *l-i SEIR* model. For example, we used *l* = 4, *i* = 10 and *α* = 0.01453 in *l-i SEIR* model for simulating daily COVID-19 cases in the US, and assumed that the first infected person in the US was exposed to COVID-19 on Feb 2, 2020. Therefore, in the conventional *SEIR* model, we let *σ* = 1/*l* = 1/4 = 0.25, *γ* = 1/*i* = 1/10 = 0.1, *β*(*t*_*n*_) = *β*_*n*_, and assumed that the first infected person in the US was exposed to COVID-19 also on Feb 2, 2020. In this way, we could immediately calculate a *y*(*t*_*n*_) curve of daily COVID-19 cases using Eq (1) in our Excel program. By visually comparing the calculated *y*(*t*_*n*_) curve with the reported daily COVID-19 cases ${\bar{y}}_{n}$ in the US on one graph in the Excel program, we found that to match *y*(*t*_*n*_) with ${\bar{y}}_{n}$, we needed to postpone the date (in initial conditions), on which the first person was exposed to COVID-19 in the US, by 7 days from Feb 2, 2020 in the *l-i SEIR* model to Feb 9, 2020 in the conventional *SEIR* model. After this was done, the *y*(*t*_*n*_) curve calculated from the conventional *SEIR* model was similar in shape to the curve of ${\bar{y}}_{n}$ reported from the US. Then by slightly regulating *β*(*t*_*n*_) day by day, we were able to match the calculated *y*(*t*_*n*_) (black dashed line in Fig 4A) with the reported ${\bar{y}}_{n}$ in the US very well. Using the same procedure, we were able to match the calculated *y*(*t*_*n*_) (black dashed line in Fig 4C) with the reported ${\bar{y}}_{n}$ in NY very well too. From Fig 4A and 4C, we can see that the *y*(*t*_*n*_) curve (black dashed line) simulated from the conventional *SEIR* model almost overlaps with the *y*_*n*_ curve (black solid line) simulated from the *l-i SEIR* model.

After this fitting process was completed, the coefficient *β*_*n*_ and *β*(*t*_*n*_) were determined, and the computation program in Excel immediately obtained and showed (*N*-*S*_*n*_), *E*_*n*_, *I*_*n*_ and *R*_*n*_ curves of the *l-I SEIR* model and (*N*-*S*(*t*)), *E*(*t*), *I*(*t*) and *R*(*t*) curves of the conventional *SEIR* model (Fig 4B and 4D). It can be seen from Fig 4B and 4D that the *I*_*n*_ curve and the *I*(*t*) curve almost overlap with each other. However, the curves of *R*_*n*_ and (*N-S*_*n*_) are more or less different from the curves of *R*(*t*) and (*N-S*(*t*)), respectively. Results in Figs 3 and 4 tell us that although we can force one of 4 variables in the conventional *SEIR* model to be very close to its corresponding variable in the *l-i SEIR* model by choosing parameters and regulating the time-dependent rate coefficients, significant calculation errors may still be generated in other 3 variables because of ignoring chronological order of individuals in the model.

**Fig 5:**
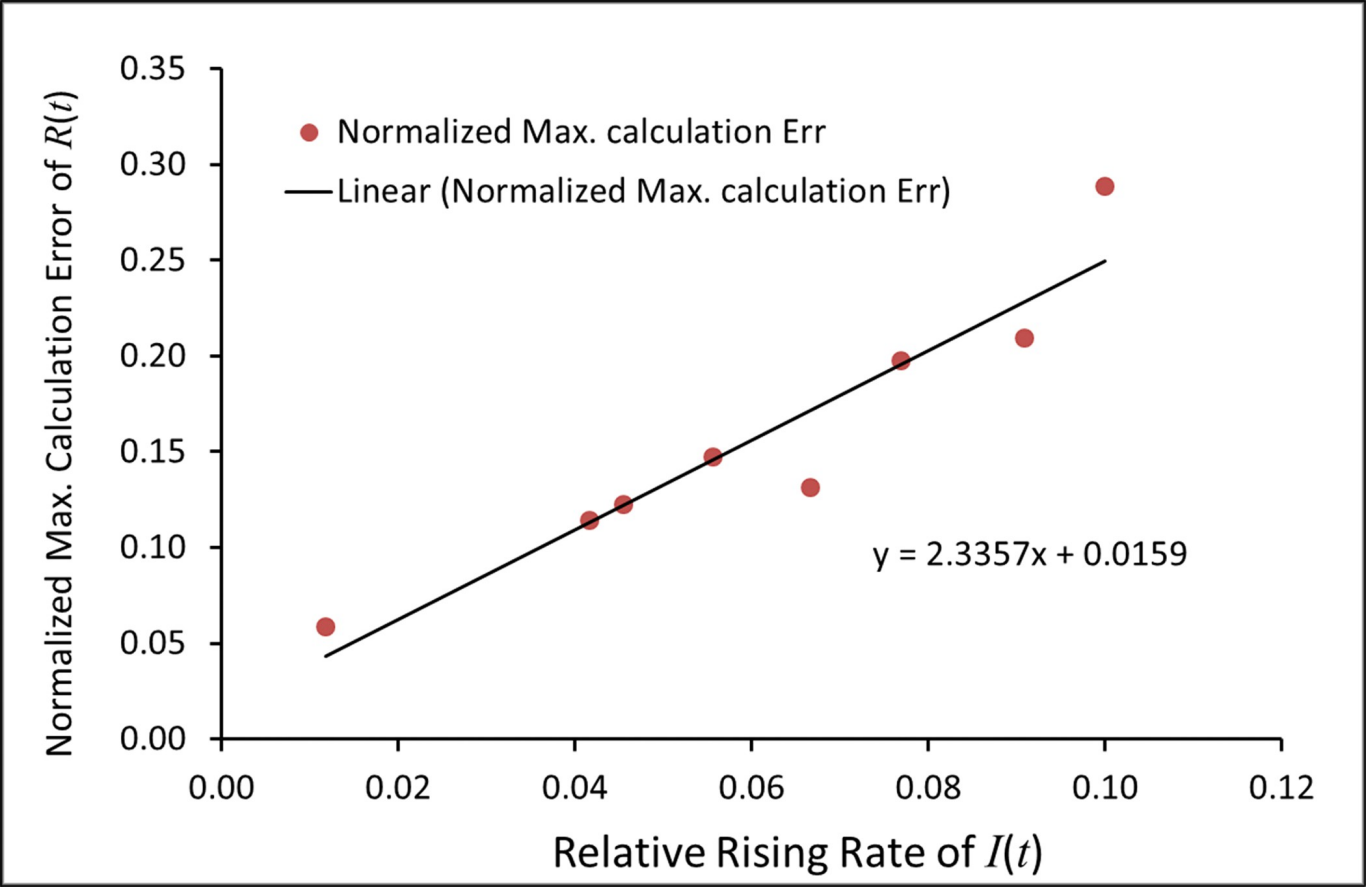
Plot of the normalized maximal calculation errors of *R*(*t*) vs. the relative rising rate of *I*(*t*) with data from Table 1.

In Fig 4D, *R*(*t*) rises earlier and faster than *R*_*n*_ before the *I*(*t*) peak, and then slower than *R*_*n*_ after *I*(*t*) peak. The bottom to top time of *I*(*t*) is 85 days (for the last wave) in Fig 4B and 22 days in Fig 4D, and so the relative rising rates of *I*(*t*) are 0.012 and 0.045 per day, respectively. Same as we have seen in Fig 3, the normalized maximal calculation error of *R*(*t*) in Fig 4 is also dependent on the relative rising rate of *I*(*t*). The relative rising rate of *I*(*t*) in Fig 4B is much smaller than that in Fig 4D, and the normalized maximal calculation error of *R*(*t*) (0.0586) in Fig 4B is also much smaller than the one (0.1223) in Fig 4D. These data obtained from Fig 4 were listed into Table 1. Using these data in Table 1, we plotted the normalized maximal calculation errors of *R*(*t*) vs the relative rising rate of *I*(*t*) (Fig 5). The result shows that the normalized maximal calculation errors of *R*(*t*) are linear with the relative rising rate of *I*(*t*) with a correlation coefficient r = 0.95.

The difference between (*N-S*(*t*)) and (*N-S*_*n*_) is similar to the difference between *R*(*t*) and *R*_*n*_ in Fig 4B and 4D. (*N-S*_*n*_) and (*N*-*S*(*t*)) are the calculated total numbers of COVID-19 infections from *l-i SEIR* model and from conventional *SEIR* model, respectively. These total numbers of infections, which include asymptomatic cases and any other infected but undetected cases, on some certain dates in the US and NY were measured by antibody tests or other methods and reported in publications or public websites. The calculated (*N-S*_*n*_) and (*N*-*S*(*t*)) in the US on September 30, 2020 are 52.5 million and 54.5 million respectively, both of which are close to 52.9 million, the real value of (*N-S*) in the US, which was estimated on the same date in a recent paper [22]. The calculated (*N-S*_*n*_) and (*N*-*S*(*t*)) in NY are 2.66 million and 2.90 million on April 23, 2020 respectively, both of which are close to 2.71 million (real value of (*N-S*) in NY) detected by antibody tests on the same date [23]. However, the conventional *SEIR* model gave relatively large calculation errors both in the US and in NY. From the above data, it is easy to calculate the relative calculation errors of (*N*-*S*(*t*)) with respect to the real value of (*N-S*) in the US on September 30, 2020 (E_US_), and with respect to the real value of (*N-S*) in NY on April 23, 2020 (E_NY_). The results show that E_US_ is 3% and E_NY_ is 7%. Correspondingly, the relative rising rate of *I*(*t*) is 0.0118 day^-1^ in the US and 0.0455 day^-1^ in NY (Table 1). These results show that the relative calculation error of (*N-S*(*t*)) increases as the relative rising rate of *I*(*t*) increases, which is consistent with the result shown in Fig 5.

Although the conventional *SEIR* model made some calculation errors, the normalized maximal calculation error made from the conventional *SEIR* model in the simulations of transmission of infectious diseases in the real world may be small. This is because the transmission rate of these infectious diseases may be small or may have been greatly slowed down by different social interventions. As shown in Fig 4B and Table 1, in the simulations of COVID-19 transmission in the US, the normalized maximal calculation error of *R*(*t*) made by the conventional *SEIR* model is only ~6% (Figs 4B & 5). However, because the normalized maximal calculation error increases if the relative rising rate of infection cases increases as we can see in Fig 4D (simulations of COVID-19 transmission in NY), Fig 3 and the data plot in Fig 5, one needs to use the conventional *SEIR* model with caution when simulating a highly transmissible infectious disease outbreak without stringent interventions to slow the spread.

In summary, the comparison of the *l-i SEIR* model with the conventional *SEIR* model was performed in the simulations of epidemic curves. It was observed that without considering the temporal heterogeneity of infected individuals, the conventional *SEIR* model couldn’t generate propagated epidemic curves when *l*>*i* like the *l-i SEIR* model, whereas the propagated epidemic curve was reported in the real world. Both theoretical analysis and simulations show that due to ignoring chronological order of infected individuals in compartments *E* and *I*, the conventional *SEIR* model overestimates (or underestimates) the transfer rate from compartment *E* to *I* and from *I* to *R* in the rising (or falling) phase of *I*(*t*). The calculation errors of variables in the conventional *SEIR* model increase with the relative rising rate of infection cases. In the simulation of daily COVID-19 cases in the US, because the rising rate is relative slow (~0.0118/day), the simulated *S*(*t*), *E*(*t*), *I*(*t*), *R*(*t*) curves from the conventional *SEIR* model are close to the simulated curves *S*_*n*_, *E*_*n*_, *I*_*n*_, *R*_*n*_ from the *l-i SEIR* model. However, in the simulation of daily cases of COVID-19 outbreak in NY with a much faster relative rising rate of infection cases (0.0455/day), there are significant differences between *R*(*t*) and *R*_*n*_, and between (*N-S*(*t*)) and (*N-S*_*n*_). These results indicate that one needs to use the conventional *SEIR* model with caution when simulating an epidemic curve with a fast rising rate, such as a highly transmissible infectious disease outbreak without stringent interventions to slow the spread.
